# Dichloroacetic acid-induced testicular toxicity in male rats and the protective effect of date fruit extract

**DOI:** 10.1186/s40360-017-0127-8

**Published:** 2017-04-22

**Authors:** Amira El Arem, Lamia Lahouar, Emna Behija Saafi, Amira Thouri, Fatma Ghrairi, Zohra Houas, Fadoua Neffati, Lotfi Achour

**Affiliations:** 1 Laboratory of Bioressources, Biology Integrative and Valorization, Higher Institute of Biotechnology of Monastir, University of Monastir, Avenue Tahar Hadded, BP 74, 5000 Monastir, Tunisia; 2 Laboratory of Histology and Cytogenetic, Faculty of Medicine, University of Monastir, Monastir, 5019 Tunisia; 3 Department of Biochemistry-Toxicology, Fattouma Bourguiba University Hospital, Monastir, Tunisia

**Keywords:** Date extract, Dichloroacetic acid, Male reproductive, Oxidative stress, Rats

## Abstract

**Background:**

The present study was designed to investigate the protective effect of aqueous date extract (ADE) against the dichloroacetic acid (DCA)-induced testicular injury in rats.

**Methods:**

Forty-eight male Wistar rats were randomly divided into six groups of eight: group I served as the control; group II was given ADE (4 ml/kg) by gavage; groups III and IV received DCA at 0.5 and 2 g/L drinking water, respectively; and groups V and VI received DCA at 0.5 and 2 g/L drinking water, respectively, before ADE administration. The experiment was performed for two months.

**Results:**

Results showed that the absolute weights of testes and epididymis were decreased following the DCA administration. The testosterone, FSH and LH levels were also decreased. Severe histopathological changes in testes were observed including degeneration of seminiferous tubules and depletion of germ cells. These changes were associated with alterations of oxidative stress markers. Levels of lipid peroxidation and SOD and CAT activities were increased, while activity of GPx and GSH levels were decreased. Pretreatment with ADE has effectively alleviated the oxidative stress induced by DCA thereby restoring these parameters to normal values.

**Conclusions:**

These results suggest that ADE has a protective effect over DCA-induced oxidative damage in rat testes.

## Background

In recent years, there has been growing concern about the deleterious effects of chemicals on developing male reproductive system thereby perturbing the testicular milieu. Despite the low oxygen tensions that characterize the testicular microenvironment, this tissue remains vulnerable to oxidative stress due to the abundance of highly unsaturated fatty acids (particularly 20:4 and 22:6) and the presence of potential reactive oxygen species (ROS)- generating system [1]. The ROS generated during normal testicular function plays an important role in regulating the function of the testes. Although ROS are known to have damaging effects, controlled, low levels of ROS play a beneficial role in normal testicular function. Conversely, increased levels of ROS can be detrimental to testicular function. To overcome this, the testis is equipped with a very potent antioxidant system that protects it from the damaging effects of ROS. The glutathione family of proteins, superoxide dismutase, catalase and several non-enzymatic antioxidants all help the testes by counteracting any oxidative impact [1]. However, overexposure to environmental toxicants has been shown to impair the pro-oxidant/antioxidant balance in the testes and thereby hamper testicular function [2].

Dichloroacetic acid (DCA) is one of the most important toxic disinfectant by-products formed during water chlorination. This compound was used in agriculture as a fungicide and consequently was detected in vegetables, fruits, and grains [3] and can be taken up into foodstuffs from the cooking water [4]. Therefore, human exposures to this compound can occur via food consumption. In another way, DCA was used clinically for therapeutic purposes, especially for the treatment of lactic acidosis in patients with mitochondrial dysfunction [5, 6]. However, long-term treatment of patients with a certain dose (10–50 mg/kg/day) of DCA was found to be associated with adverse effects that included mild liver dysfunction, hypoglycemia and changes in the central and peripheral nervous system [5, 6].

Furthermore, there is an extensive and consistent data base demonstrating the reproductive toxicity of chronic DCA treatment in male including polyneuropathy and testicular degeneration [7–10].

Several studies were carried out to evaluate the potential role of antioxidants, such as synthetic or isolated from medicinal plants, for the protection of cells against oxidative damage and reproductive toxicity due to environmental toxins [11–15]. These substances have shown their effectiveness to attenuate the oxidative damage, lipid peroxidation, and toxic effects produced in a wide array of systems, organs, and tissues.

Date palm (*Phoenix dactylifera* L.) is one of the oldest trees dating from 6000 years. The various parts of this plant are widely used in traditional medicine for the treatment of various disorders [16, 17]. Date fruits are the most commonly used part due to their richness in several nutrients (dietary fibers, sugars, vitamins, proteins, fat), beside to their dietary antioxidants (flavonoids, phenolic acids, sterols, anthocyanins, carotenoids, tanins, selenium, zinc, magnesium). These fruits are consumed at any of the three major stages of maturity such as besser or khalal (fresh, hard ripe, color stage), rutab (crisp to succulent or ripe stage), or tamr (soft pliable, full ripe stage). The information accrued in the past four decades suggest that dates possess diverse medical uses including anti-hyperlipidemic, anti-cancer, anti-inflammatory, gastroprotective, hepatoprotective and nephroprotective activities and thereby serving as an important healthy food in the human diet [16]. In traditional medicinal practices, dates are considered as tonic and aphrodisiac [18]. Despite several studies have tested the repro-protective effect of date pits and pollen extracts and aqueous fruit extract of mature date fruits [19–23], no study has been reported in the preventive effect of date fruit at besser stage.

For this reason, this study was carried out to determine the effect of subchronic exposure to two carcinogenic doses (0.5 and 2 g/l) [24, 25] of dichloroacetic acid on the reproductive system of male rats and to assess whether these effects can be ameliorated by pretreatment with aqueous date extract of date fruit, at besser stage.

## Methods

### Date palm fruit extract preparation

The Degla variety was collected from the station of Souk Lahad (Kebili, Tunisia) at besser stage of maturation. The flesh was manually separated from the pits, soaked in distilled water (1:3 ratio, weight to volume) and kept for 48 h at a temperature of 4 °C. Then the mixture was centrifuged at 4 °C for 20 min at 4000 × *g*, and the supernatant was collected [26].

### Experimental design

Forty-eight male Wistar rats weighing 180–200 g were obtained from Central Pharmacy of Tunis, Tunisia. Animals were divided into six equal groups of eight each and were housed under standard laboratory conditions with a 12-h light–dark cycle at constant temperature (22 ± 2 °C) and humidity (55 ± 5%) and kept on commercial diet and tap water provided *adlibitum*. The animals were handled according to the guidelines of the Tunisian Society for the Care and Use of Laboratory Animals, and the study was approved by the University of Tunisia Ethical Committee (approval number: FST/LNFP/Pro 152012).

After the acclimatization period, the groups were assigned at random to one of the following treatments: group 1 served as control receiving saline (0.9%), group 2 received a daily oral dose (4 ml/kg) of aqueous date extract (ADE), groups 3 and 4 were orally treated respectively with dichloroacetic acid at 0.5 g/l (DCAC1) and DCA at 2 g/l (DCAC2) as drinking water, while groups 5 and 6 received ADE plus DCAC1 or DCAC2. All the animals were observed daily for the presence of clinical signs of toxicity during the two months of the study.

### Sample collection

After 2 months of the treatment period, rats were anesthetized by inhalation of diethyl ether, and blood was drawn by cardiac puncture and collected into silicon disposable glass tubes with EDTA as an anticoagulant. Tubes were centrifuged at 4000 *g* for 15 min at 4 °C. The plasma samples were stored at−20 °C in aliquots until analysis. Testes and epididymis were excised immediately washed with an ice-cold physiologic saline solution (0.9%, *w*/*v*), blotted dry and weighed. About 1 g of one testis was cut into small pieces, homogenized with an Ultra Turrax homogenizer T25 (Cole-Parmer) in 3 volumes of ice-cold appropriate buffer (TBS, pH 7.4) and centrifuged at 9000 *g* for 15 min at 4 °C. Supernatants were collected, aliquoted and stored at−80 °C until use for enzyme assays and lipid peroxidation. Bradford’s [27] method was used to determine the protein content.

### Plasma levels of FSH, LH and testosterone

The plasma FSH and LH were determined by radioimmunoassay (RIA) using reagents from a commercial kit (SBTesto,CIS BioInternational, Gif-sur-Yvette, France). Plasma levels of testosterone were determined by a competitive radioimmunoassay kit (Immunotech, Beckman Coulter, France) using 125I-labeled testosterone analog as the radioactive marker. The monoclonal antibody used in the immunoassay was highly specific for testosterone. The assay was performed as per the manufacturer’s instructions. The amount of radioactive exogenous testosterone bound to the antibody is inversely proportional to the concentration of the endogenous testosterone present.

### Antioxidant status and lipid peroxidation

Superoxide dismutase (SOD) activity was assayed spectrophotometrically as described by Beyer and Fridovich [28]. This method is based on the capacity of SOD to inhibit the oxidation of nitrobluetetrazolium (NBT). One unit of SOD represents the amount of enzymes required to inhibit the rate of NBT oxidation by 50% at 25 °C. The activity was expressed as units/mg protein.

Catalase (CAT) activity was measured by the UV colorimetric method of Aebi [29] using H_2_O_2_ as substrate. One unit of CAT activity is equal to the μmol of H_2_O_2_ destroyed/min/mg proteins.

Glutathione peroxidase (GPx) activity was assayed according to the method of Flohe and Gunzler [30]. The activity was expressed as μmol of GSH oxidized/min/mg of protein, at 25 °C.

Reduced glutathione (GSH) level was measured colorimetrically as protein-free sulfhydryl content using 5,5-dithiobis-2-nitrobenzoic acid (DTNB) according to the method of Sedlak and Lindsay [31]. The values were expressed as μmol/mg proteins.

Lipid peroxidation (LPO) was determined indirectly by measuring the production of malondialdehyde (MDA) in the testes extract according to the method of Buege and Aust [32] based on TBA reactivity. The absorbance was measured at 530 nm and was proportional to the amount of TBARS formed. The results were expressed as nmol MDA equivalents/mg protein.

### Histological examination

One testis of each rat was removed and quickly fixed in 10% buffered-neutral formalin, routinely processed, embedded in paraffin and sections of 5 μm thick were cut. Hematoxylin and eosin (H&E) were used for staining [33]. The histological examination was observed at the light microscopic level. The sections were analyzed by a certified pathologist ignoring the sample assignments to experimental groups. A minimum of three fields of each testis slide were morphologically evaluated.

### Statistical analysis

All experimental data were expressed as mean ± standard deviation. The statistical analysis was done by one-way analysis of variance (ANOVA) followed by Duncan’s test using SPSS 11.0 for Windows. Statistical significance was set at *p* < 0.05.

## Results

### Testes and epididymis weights

**Fig. 1:**
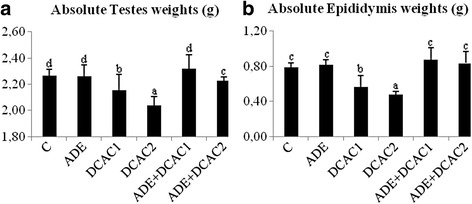
Effect of ADE on reproductive organs weights ((**a**) testis and (**b**) epididymis) in male rats treated with two different doses of DCA (0.5 and 2g/l). Different letters (a-d) indicate significant differences between groups at *p*<0.05 by Duncan’s test. ADE: aqueous date extract, DCAC1: dichloroacetic acid at 0.5g/l, DCAC2: dichloroacetic acid at 2g/l

Weights of testes and epididymis, expressed as absolute body weight, in rats after DCA administration was found to be significantly decreased, compared with the control group (Fig. 1). This decrease was more induced with the higher dose of DCA (2 g/L). However, pretreatment with the ADE restored the absolute testicular and epididymis weights as compared to DCA intoxicated rats.

### Serum FSH, LH and testosterone levels

The effects of ADE on plasma FSH, LH and testosterone levels are shown in Table 1. DCA administration in rats for 2 months induced significant dose-dependent decreases (*p* < 0.05) in these hormonal levels as compared to control group. Alterations of these hormones were significantly reversed (*p* < 0.01) by pretreatment with the ADE comparatively to DCA intoxicated groups. No significant (*p* > 0.05) changes were observed in rats treated with the ADE alone.

**Table 1:** Effect of ADE on plasma levels of FSH, LH and testosterone in male rats treated with two different doses of DCA

	Testosterone (ng/ml)	FSH (mlU/ml)	LH (mlU/ml)
C	3.45 ± 0.10c	3.20 ± 0.02c	1.29 ± 0.01c
ADE	3.46 ± 0.07c	3.19 ± 0.01c	1.28 ± 0.01c
DCAC1	2.30 ± 0.28b^a^	2.94 ± 0.09b	1.18 ± 0.01b
DCAC2	1.81 ± 0.17a^a^	2.19 ± 0.05a^a^	0.80 ± 0.01a^a^
ADE+ DCAC1	3.30 ± 0.32c	3.17 ± 0.02c	1.28 ± 0.01c
ADE+ DCAC2	2.96 ± 0.03c	3.08 ± 0.03bc	1.22 ± 0.01c

Values are mean ± SD of eight rats in each group. Different letters (a-c) indicate significant differences between groups at *p* < 0.05. C: control, ADE aqueous date extract, DCAC1: dichloroacetic acid at 0.5 g/l, DCAC2: dichloroacetic acid at 2 g/l

^a^Comparison of control and other groups

### Antioxidant status and lipid peroxidation levels

Results of testicular antioxidant status and lipid peroxidation have been depicted in Table 2. The exposure of rats to DCA for 2 months caused a significant (*p* < 0.05) dose-dependent increase in testes SOD and CAT activities and LPO and a significant (*p* < 0.05) dose-dependent decrease of GPx activity and GSH levels compared with those of control group. These alterations were more marked with the higher DCA dose (approximately by 2 fold). However, pretreatment with ADE improved significantly (*p* < 0.05) these parameters. ADE alone did not produce any significant changes in enzymes antioxidant activity, LPO and GSH levels.

**Table 2:** Effect of ADE on testicular antioxidant enzymes activities and GSH and LPO levels in male rats treated with two different doses of DCA

	CAT (μmolH_2_O_2_/min/mg proteins)	SOD (U/mg proteins)	GPx (μmol GSH oxidized/min/mg proteins)	GSH (μmol/mg proteins)	LPO (nmol MDA/mg proteins)
C	191.47 ± 8.95a	3.03 ± 0.29a	11.36 ± 0.93c	0.36 ± 0.01c	1.08 ± 0.03a
ADE	184.62 ± 11.14a	3.09 ± 0.35a	11.61 ± 0.80c	0.37 ± 0.04c	1.08 ± 0.07a
DCAC1	277.48 ± 12.17c^a^	4.13 ± 0.53b^a^	10.81 ± 0.79b	0.26 ± 0.04b^a^	1.84 ± 0.11c^a^
DCAC2	370.04 ± 12.75d^a^	5.31 ± 0.46c^a^	9.37 ± 0.76a^a^	0.18 ± 0.03a^a^	2.39 ± 0.16d^a^
ADE + DCAC1	192.28 ± 10.36a	3.31 ± 0.32a	11.62 ± 0.58c	0.36 ± 0.05c	1.14 ± 0.06a
ADE + DCAC2	215.79 ± 13.09ab	3.99 ± 0.50ab	11.16 ± 0.99c	0.30 ± 0.04bc	1.26 ± 0.03ab

Values are mean ± SD of eight rats in each group. Different letters (a-c) indicate significant differences between groups at *p* < 0.05. C: control, ADE aqueous date extract, DCAC1: dichloroacetic acid at 0.5 g/l, DCAC2: dichloroacetic acid at 2 g/l, LPO: lipid peroxidation

^a^Comparison of control and other groups

### Histological examination

**Fig. 2:**
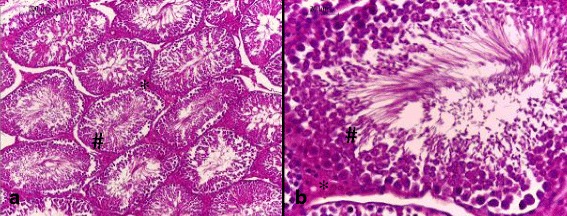
Testis section of control and ADE treated groups showing (**a**) normal histological architecture pattern of seminiferous tubules with (**b**) normal germ cells (**#**) and interstitial connective tissue (*) (H&E, (**a**) 100× and (**b**) 400×)

**Fig. 3:**
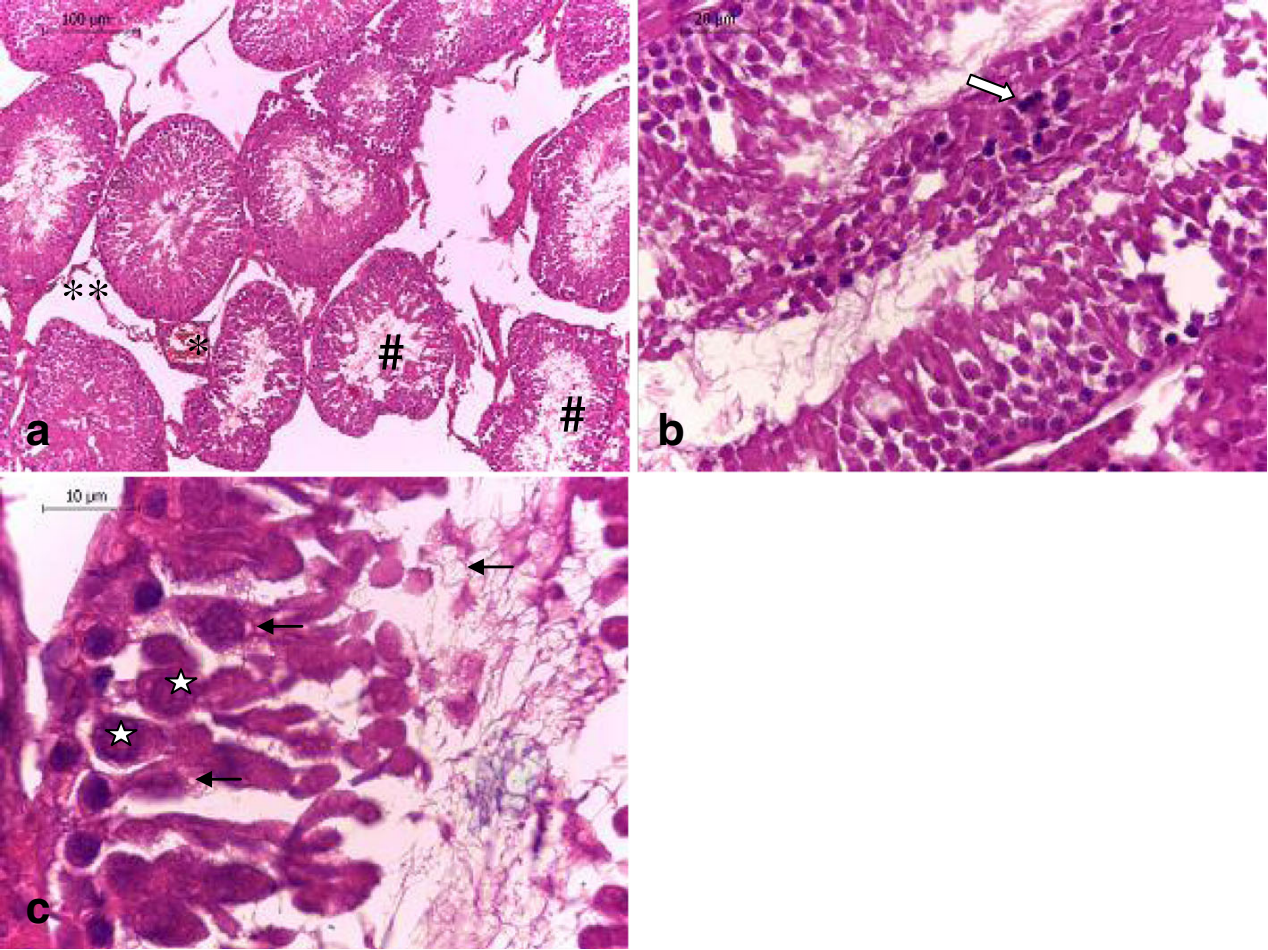
Testis section of rats treated with DCA at 0.5 g/l showing (**a**) congestion of blood vessels (*), tubular atrophy with extensive degeneration in some tubules and depletion of germ cells (#) and Leydig cells (**), (**b**) necrosis (⇒), and (**c**) vacuolation of epithelium cells () and disintegration of the spermatogenic cells () (H&E, (**a**) 100×, (**b**) 400× and (**c**) 1000×)

**Fig. 4:**
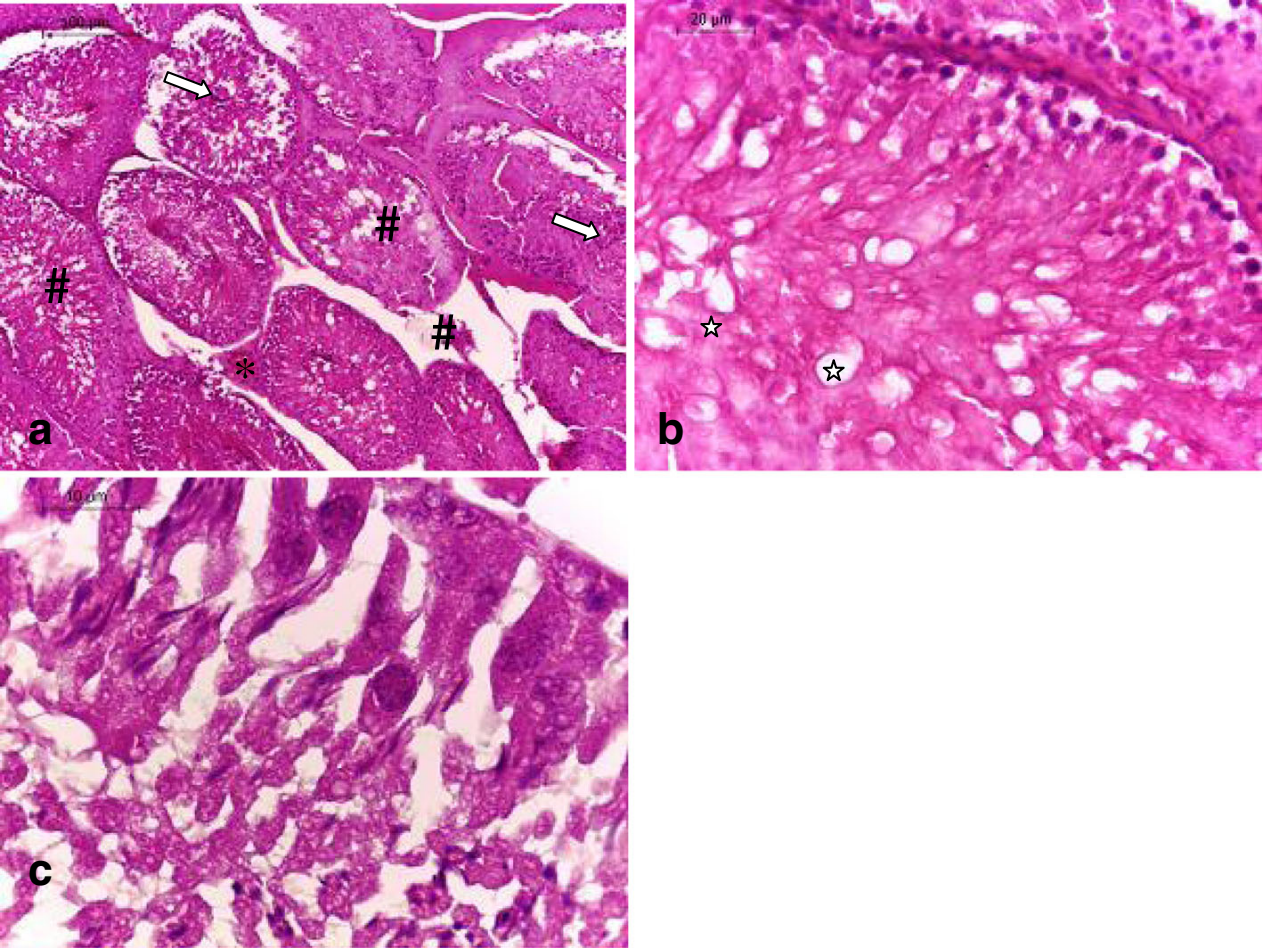
Testis section of rats treated with DCA at 2 g/l showing marked disturbances of the normal architecture of the testicular tissues; (**a**) congestion of blood vessels (), hyperplasia of seminiferous epithelium and Leydig cells (#), necrotic cells in the lumina (⇒), (**b**) cytoplasmic vacuolation and edema (), and (**c**) necrotic and vacuolar degeneration of spermatogonia, spermatocyte I and sertoli cells was evident (H&E, (**a**) 100×, (**b**) 400× and (**c**) 1000×)

**Fig. 5:**
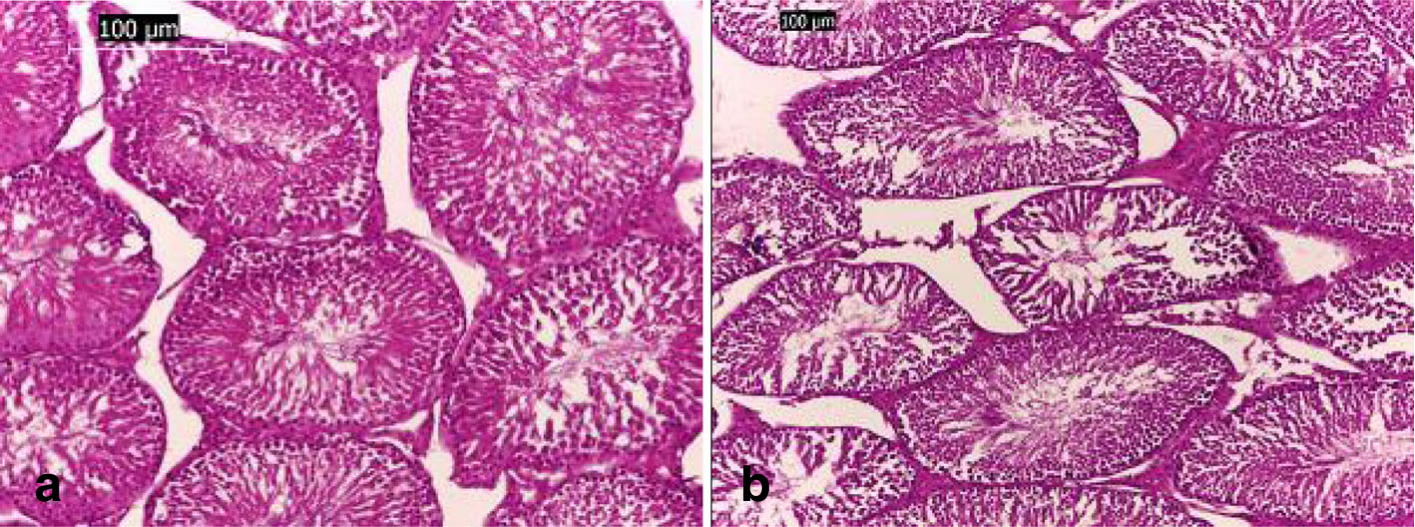
Testis sections of rats treated with (**a**) ADE and DCA at 0.5g/l showing significant improvement of histological architecture with normal seminiferous and sertoli cells, and (**b**) those treated with ADE and DCA at 2g/l showing mild degenerative changes of seminiferous tubules, mild congestion and edema (H&E, 100×)

Light microscopic examinations of the testicular sections of the control rats and those receiving ADE alone showed normal histoarchitecture that consisted of uniform, well-organized seminiferous tubules with complete spermatogenesis and normal interstitial connective tissue (Fig. 2a, b ). The testicular tissue of rats received DCA alone showed degenerative changes of the majority of the seminiferous tubules (Figs. 3 and 4). Treatment of rats with DCA at 0.5 g/l (Fig. 3 a, b, c) caused extensive degeneration in some tubules and depletion of germ cells and Leydig cells, vacuolation of epithelium cells and disintegration of the spermatogenic cells, and necrosis in some other seminiferous tubules. Regarding the interstitium, there were dilation and congestion of the interstitial blood vessels and mild edema. These alterations were more accentuated in rats treated with DCA at 2 g/L (Fig. 4). These rats were characterized by reduced and disorganized seminiferous tubules, distorted basement membrane, greatly depleted of germ cells and incomplete spermatogenesis. Moreover, the seminiferous tubules were almost devoid of spermatids and spermatozoa and showed cytoplasmic vacuolation. Vacuolar degeneration of spermatogonia, spermatocyte I and Sertoli cells was evident. Necrotic cells in the lumina of most seminiferous tubules were abundant. Marked dilation and congestion of blood vessels were noticed in the interstitial spaces. Hyperplasia of Leydig cells was detected in the interstitial tissue; there was crowding of Leydig cells forming dense clumps that surround most of the seminiferous tubules. Conversely, testes of rats pretreated with ADE revealed a marked repairing of testicular abnormalities induced by DCA at 0.5 g/l near to control group (Fig. 5a). However, those pretreated with ADE and receiving DCA at 2 g/l (Fig. 5b) showed mild interstitial edema and slight vacuolization of the germinal epithelial cells. A few numbers of seminiferous tubules had shown necrotic germinal epithelium in their lumina. There was a marked improvement of spermatogenesis, evidenced by the presence of elongated spermatids and spermatozoa in the majority of seminiferous tubules.

## Discussion

The subchronic treatment of rats with DCA at both concentrations of 0.5 and 2 g/l (corresponding to 33.78 and 131.24 mg/kg/day, respectively) during two months induced dose-dependent decreases in absolute testes and epididymis weights. These results are in agreement with those found in previous studies [9, 10, 34, 35] using different concentrations of DCA during different treatment periods in rat and dogs. The weight of the testicles is largely dependent on the mass of the differentiated spermatogenic cells. Thus a reduction in its weight might be due to the decreased number of germ cells and inhibition of spermatogenesis and steroidogenic enzyme activity [12, 36]. In fact, different studies [7, 9, 10] demonstrated that repeated administration of DCA to animal induced, in a dose-dependent way, testicular damage associated with spermatogenic damage and germinal epithelial degeneration. These findings are confirmed in our study by histopathological lesions. A previous study has shown that the pathological changes of seminiferous epithelium may cause the disruption of Sertoli and germ cells, which results in impaired spermatogenesis and may also lead to germ cell loss [37].

It is known that normal spermatogenesis depends on the testosterone secretion and gonadotropic hormonal action of the pituitary gonadotropins such as luteinizing hormone and follicle stimulating hormone. LH stimulates testosterone production and secretion by Leydig cells, while FSH acts directly on the seminiferous tubules [38, 39]. Contrary to the study of Linder et al. [10] which reported no effect on testosterone levels in rats intoxicated for 14 consecutive days at 18 to 1440 mg kg/b.w, we observed a significant decrease of testosterone levels at 131.24 mg/kg b.w dose of DCA after 2 months of intoxication. These results are similar to those found in rats treated by four trihalomethanes, other water disinfection by-products [40]. These results are also confirmed by the histopathological changes in the number and structure of Leydig cells in DCA-intoxicated rats. Sanghamitra et al. [41] suggested that degeneration of Leydig cell resulted in decreased synthesis of testosterone, which in turn disturbs the process of spermatogenesis. Previous studies have shown that exposure to environmental contaminants adversely affects testicular function by decreasing pituitary LH secretion and reducing Leydig cell steroidogenesis [42, 43]. In the present study, the subchronic DCA-intoxication induced significant dose-dependent depletion in FSH and LH levels, mostly marked in rats intoxicated with the higher DCA dose (2 g/l). These results are in agreement with those found by Klinefelter et al. [44] in rats treated with bromochloroacetic acid. The observed reduction in plasma LH and FSH levels in DCA-exposed rats suggest that DCA perturbs anterior pituitary hormone synthesis and secretion.

In our previous studies [45, 46] we have shown that DCA induced oxidative stress in liver and renal tissues by affecting endogenous antioxidant defense enzymes and lipid peroxidation. In the same way, in this study, we found that DCA induced oxidative stress in testicular tissues illustrated by an alteration in antioxidant enzymes activity and in lipid peroxidation level beside a decrease in GSH level. Antioxidant enzymes represent a defense mechanism, and are responsible for detoxification of reactive oxygen species (ROS). The SOD–CAT system provides the first defense against oxygen toxicity. SOD is considered to be one of the most active enzymes, its activity is sufficient for the dismutation of superoxide anions to hydrogen peroxide (H_2_O_2_) produced during oxidative stress in cells [47]. The elimination of H_2_O_2_ is either effected by CAT or GPx, with the latter playing predominating role in the testes [48]. Induction of SOD could occur during high production of superoxide anion. Therefore, an increase in the SOD activity indicates an increase of O˙_2_^−^ production. Also, the elevated activity of CAT may due to the adaptive response to the generated free radicals. Thus increased activities of SOD and CAT may serve as protective responses to eliminate reactive free radicals [49]. GPx is involved in catalyzing the reduction of H_2_O_2_ at the expense of reduced GSH [50]. The level of GPx in the DCA-intoxicated rats, namely those receiving the higher dose, was depleted which can be attributed to either increased H_2_O_2_ generation or decreased GSH concentration. Decrease in GSH level as observed in the present study may be due to increased utilization of GSH for metabolism of lipid hydroperoxides by GPx or interaction of GSH with free radicals [13]. Thiol-based antioxidant system is the primary line of defense against oxidative stress. The decreased concentration of GSH increases the sensitivity of organ to oxidative and chemical injury and is one of the primary factors which permit lipid peroxidation. In agreement with this hypothesis is the increase in MDA levels in rats’ testes observed in this study. MDA is a major oxidation product of peroxidized polyunsaturated fatty acids and increased MDA content is an important indicator of lipid peroxidation [51]. Therefore the increase in lipid peroxidation is the reflection of damage caused to the membranous structures. These disturbances in antioxidant enzyme and non-enzymatic systems and increases in lipid peroxidation in testes of rats intoxicated with DCA in the present study are in agreement with those induced by different other environment toxicants [15, 52–55]. Histoarchitecture changes as seen in this study are supported by the significant changes seen in the enzymes and non-enzyme antioxidant defense systems as well as lipid peroxidation, which reflect increased ROS production. Nagda and Bhatt [13] suggested that enhancement of ROS production in the testes may further damage vital components of the cell, like nucleic acids and proteins which further lead to oligozoospermia and abnormal spermatozoa. These findings support the results from other reports that DCA can seriously alter the testicles and reproductive tract in male rats [7, 9, 10].

Several studies have shown the ability of mature date fruits extract to increase sperm count, sperm motility, and viability [56] and to enhance spermatogenesis and increased the concentration of testosterone, FSH and LH [22] in intoxicated male rats. However, no study has been reported on the repro-protective effect of these fruits at besser stage of maturation. In the present study, the pretreatment with the ADE restored the testes and epididymis weights and histopathological alterations caused by DCA and TCA, in addition to retaining the control values of oxidative stress markers. The observed therapeutic potency of ADE might be due to several contributing factors, primarily including the mineral (zinc, selenium, copper, iron, calcium, cobalt, magnesium, manganese) and the vitamins (A, B, C) composition of these fruits [16]. Zinc is an acknowledged antioxidant factor that as well as being a core constituent of free radical scavenging enzymes such as SOD and a recognized protector of sulfhydryl groups is also thought to impair lipid peroxidation by displacing transition metals such as iron and copper from catalytic sites [57]. Selenium is an antioxidant trace element that is involved in the metabolism of free radicals and other substances produced by the oxidation of lipids in cell membranes. It also plays a role in metabolism in the liver and contributes to the maintenance of skeletal and cardiac muscles and sperm. Selenium is also a component of the majority of glutathione peroxidase antioxidant enzymes that are important in supporting the testicular function [1]. Vitamin C (ascorbic acid) contributes to the support of spermatogenesis at least in part through its capacity to reduce α-tocopherol and maintain this antioxidant in an active state [58]. Vitamin C is itself maintained in a reduced state by a GSH-dependent dehydroascorbate reductase, which is abundant in the testes [59]. Deficiencies of vitamins C lead to a state of oxidative stress in the testes that disrupts both spermatogenesis and the production of testosterone [60]. The second mechanism by which aqueous date fruit extract protect testicles may be attributed to their richness in various polyphenolic compounds namely the flavonoids that are highly efficient against ROS-mediated injury [61]. The quercetin, a bioactive flavonoid component, of date fruit at this besser stage [62] may be one of the responsible of this observed protective effect. It was suggested that quercetin acts as an antioxidant by inhibiting oxidative enzymes such as xanthine oxidase, lipoxygenase, and NADPH oxidase. Inhibition of these enzymes is also responsible for the attenuation of oxidative stress as they play key roles in the initial process of free radical-induced cellular damage [63]. It was recently reported that quercetin improved the antioxidants status in rats’ testes and prevented spermatogonial cells from oxidative stress damage caused by reproductive toxicants [64, 65]. In other way, a study reported by Michael et al. [66] showed that treatment of alloxan diabetic male rats with two diosmetin glycosides flavonoid compounds (diosmetin 7-O-β-L-arabinofuranosyl (1–2) β-D-apiofuranoside and diosmetin 7-O-β-D-apiofuranoside) isolated from the epicarp of date fruits, increased significantly serum testosterone level accompanied with highly significant decrease in total and prostatic acid phosphatase activities. Moreover, date fruit contains melatonin [67]. Melatonin is soluble in both lipid and aqueous environments and can readily cross the blood‑testes barrier to protect the germinal epithelium [1]. A study reported by Rao & Bhatt [68] showed that melatonin significantly protects the gonadal function against fluoride induced testicles toxicity in rat model. This effect was attributed to its free-radical scavenging actions beyond its stimulatory effects on antioxidant enzyme systems.

## Conclusion

The present study indicates that low and high doses of DCA induced testicular toxicity in male rats. The pretreatment of DCA-intoxicated groups with ADE restored the lipid peroxidation, the antioxidant enzymes’ status and the histoarchitecture of DCA-damaged testes. The protective effect of ADE may be due to its antioxidant property and detoxification capacity.

## Abbreviations

*ADE*: Aqueous date extract
*CAT*: Catalase
*DCA*: Dichloroacetic acid
*FSH*: Follicle-stimulating hormone
*GPx*: Glutathione peroxidase
*GSH*: Glutathione
*LH*: Luteinizing hormone
*LPO*: Lipid peroxidation
*MDA*: Malondialdehyde
*NBT*: Nitrobluetetrazolium
*ROS*: Reactive oxygen species
*SOD*: Superoxide dismutase
